# The effect of dietary restriction on reproduction: a meta-analytic perspective

**DOI:** 10.1186/s12862-016-0768-z

**Published:** 2016-10-07

**Authors:** Joshua P. Moatt, Shinichi Nakagawa, Malgorzata Lagisz, Craig A. Walling

**Affiliations:** 1 Institute of Evolutionary Biology, School of Biological Sciences, University of Edinburgh, Ashworth Labs, Kings Buildings, Edinburgh, EH9 3JT UK; 2 Evolution & Ecology Research Centre and School of Biological, Earth and Environmental Sciences, University of New South Wales, Sydney, NSW 2052 Australia; 3 Diabetes and Metabolism Division, Garvan Institute of Medical Research, Sydney, NSW 2010 Australia

**Keywords:** Nutrition, Breeding, Life history trade-off, Meta-analysis, Systematic review

## Abstract

**Background:**

Dietary restriction (DR), a reduction in the amount of food or particular nutrients eaten, is the most consistent environmental manipulation to extend lifespan and protect against age related diseases. Current evolutionary theory explains this effect as a shift in the resolution of the trade-off between lifespan and reproduction. However, recent studies have questioned the role of reproduction in mediating the effect of DR on longevity and no study has quantitatively investigated the effect of DR on reproduction across species.

**Results:**

Here we report a comprehensive comparative meta-analysis of the effect of DR on reproduction. In general, DR reduced reproduction across taxa, but several factors moderated this effect. The effect of DR on reproduction was greater in well-studied model species (yeast, nematode worms, fruit flies and rodents) than non-model species. This mirrors recent results for longevity and, for reproduction, seems to result from a faster rate of decline with decreasing resources in model species. Our results also suggested that not all reproductive traits are affected equally by DR. High and moderate cost reproductive traits suffered a significant reduction with DR, but low cost traits, such as ejaculate production, did not. Although the effect of DR on reproduction was stronger in females than males, this sex difference reduced to near zero when accounting for other co-factors such as the costliness of the reproductive trait. Thus, sex differences in the effect of DR on longevity may be due to a failure to expose males to as complete a range of the costs of reproduction as females.

**Conclusions:**

We suggest that to better understand the generality of the effect of DR, future studies should attempt to address the cause of the apparent model species bias and ensure that individuals are exposed to as many of the costs of reproduction as possible. Furthermore, our meta-analytic approach reveals a general shortage of DR studies that record reproduction, particularly in males, as well as a lack of direct side-by-side comparisons of the effect of DR on males and females.

**Electronic supplementary material:**

The online version of this article (doi:10.1186/s12862-016-0768-z) contains supplementary material, which is available to authorized users.

## Background

Dietary restriction (DR), defined as a reduction in food intake without malnutrition [1, 2], has been shown to extend lifespan and protect against age related diseases across a range of studies (see [1, 3] for current reviews). The majority of studies examining DR use one of five laboratory model species: *Saccharomyces cerevisiae* [4], *Caenorhabditis elegans* [5], *Drosophila melanogaster* [6], *Mus musculus* and *Rattus norvegicus* [7], hereafter referred to as “model species” (see [1]). The taxonomic diversity of these model species and the fact that the effect of DR is reproducible in other, less commonly studied taxa (e.g. Primates [8]; arachnids [9]; fish [10]), has been used to suggest that the effect of DR on longevity is underpinned by an evolutionarily conserved mechanism and may thus have application to humans [3]. However, a recent meta-analysis has demonstrated that dietary restriction is nearly twice as effective at extending lifespan in the five model species as it is in non-model species [1]. Such an overarching pattern questions the taxonomic generality of this effect and thus the suggestion of an evolutionarily conserved mechanism.

The dominant evolutionary explanation of the effect of DR on longevity is based on the disposable soma theory of ageing [11, 12]. Under DR, it is hypothesised that organisms should reallocate resources away from reproduction to somatic maintenance (and thus survival) in order to increase the chance of surviving the period of resource limitation, and thus reproducing when more favourable conditions return [12]. A key prediction therefore is that increased longevity is a direct consequence of reduced reproduction. This prediction initially appears well supported; both among and within species fecundity is generally negatively correlated with longevity [13] and many studies cite a negative effect of DR on reproduction. However, close inspection reveals that these citations generally involve one of three studies: two using *D. melanogaster* [14, 15], cited 345 and 362 times respectively, (Google Scholar, accessed 07/09/2016) and the third study using rats [16], cited 89 times (Google Scholar, accessed 07/09/2016). More recently, studies have questioned the generality of the longevity-reproduction trade-off underlying the effect of DR, with some data suggesting that longevity and reproduction can be uncoupled [17, 18]. In *D. melanogaster*, for example, significant lifespan extension through DR was achieved in females that were incapable of vitellogenisis or had impaired ovarian activity and could not produce eggs [17]. Furthermore, many studies of DR fail to detect a decrease in reproduction, an increase in longevity or both [19–21]. These exceptions and the fact that a small number of studies using model species (where the DR effect on longevity is known to be greater [1]) are highly cited to support the longevity-reproduction trade-off underlying DR, suggest that an investigation into the generality of the effect of DR on reproduction is warranted.

One common observation is sexual dimorphism in the response to DR, with lifespan extension greater in females than in males [22–24]. Although direct comparisons between the sexes within the same study are rare (see below and [22]), the generality of this pattern has been supported by a recent meta-analysis showing a 20 % greater lifespan extension under DR in females than males [1]. An intuitive explanation is that females invest more in reproduction than males. However, although this may be true on a per-gamete basis, males invest heavily in reproduction via other avenues e.g. courtship, intra-male competition and territory defence, such that on average the net costs of reproduction must be equal in males and females [25, 26]. The fact that male costs of reproduction are generally not associated with gamete production may mean that males have not been exposed to the full costs of reproduction in current DR studies. In many studies males and females are kept separately and often in isolation (e.g. [21, 23, 27, 28]), and thus males do not experience the costs associated with e.g. courtship and competition. Thus, the sex difference in the effect of DR may be a result of sex differences in the costs of reproduction experienced. If this hypothesis is correct, we would predict a sex difference in the effect of DR on reproductive traits, with DR having more of an effect on higher cost traits. We expect that taking this into account will remove any sex difference in the effect of DR on reproduction.

Another area to explore is how reproductive decline changes with increasing levels of DR. The disposable soma theory of DR predicts an initially linear decrease in reproduction with decreasing resources. However, at very low levels of resources survival becomes unlikely and some degree of terminal investment is predicted [12], resulting in a decrease in the rate of reproductive decline. Recently an alternative to the disposable soma theory of DR has proposed that the response to DR evolved to minimise the loss of reproduction through upregulation of cell recycling mechanisms such as apoptosis and autophagy [29]. We suggest that this theory also predicts a non-linear reproductive decline with increasing DR. However, in this case the decrease in reproduction should be initially shallow, as cell recycling copes with small reductions in resources via recapture of some internal resources; a faster rate of decline should be observed at higher restriction levels. By examining the pattern of reproduction across levels of DR we can test these two hypotheses.

In this study we therefore attempt to address a number of issues surrounding the effect of DR on reproduction using a systematic review and meta-analysis. This method allows us to combine data from a diverse range of species, across a number of different studies. We can then highlight any general trends in the effect of DR on reproduction, whilst controlling for species-specific and study-specific effects. The specific aims of this paper are thus to investigate: (1) the generality of the effect of DR on reproduction; (2) whether, as for longevity, the effect of DR on reproduction is stronger in model than non-model species; (3) whether, as for longevity, there are sex differences in the effect of DR on reproduction; (4) whether these sex differences can be explained by the likely costliness of the reproductive traits investigated; and (5) the shape of reproductive decline with increasing restriction levels. More generally, this study aims to provide a quantitative summary of the current understanding of the effect of DR on reproduction and thus highlight areas where our knowledge is lacking and further research would be valuable.

## Methods

### Data collection and effect size extraction

**Fig. 1:**
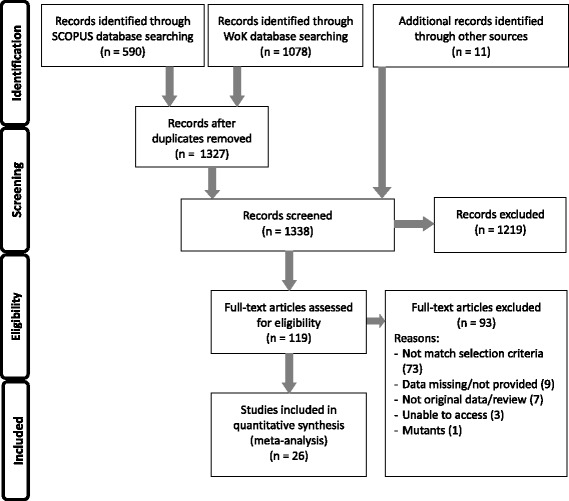
PRISMA flow diagram of data collection. The number of papers identified initially through key word searching is shown in the identification boxes. The number of papers excluded is shown for each stage of screening. Reasons for exclusion are given for papers that made it to final eligibility screening

Detailed descriptions of data collection and analysis are given in Additional file 1: Dialog S1. Briefly, data were collected through a search of *ISI Web of Science* and *Scopus* using the search strings ‘diet*/calor* + restriction + reproduction/fertility/fecundity’. Backward and forward searching was carried out to identify additional papers that were missed in the main database search and the authors’ own literature collections on the subject were considered. These searches yielded 1679 papers (Fig. 1), of which 26 reported some measure of reproduction in treated (DR) and control females or males and matched the additional selection criteria (see Additional file 1: Dialog S1 for details). This is perhaps a surprisingly low number of studies given the interest in DR and longevity, highlighting the paucity of studies that also collect data on reproduction. Full details for why studies were rejected are provided in data S3 provided with our data supplement on dryad (doi:10.5061/dryad.3fc02), but a number of studies were rejected as a result of not applying DR consistently across life. It is worth noting that different selection criteria would result in a different selection of studies being included and may affect our results, but we do not think our selection criteria were overly restrictive or would cause any particular bias. The 26 studies used covered 21 species (see Additional file 1: Figure S1 for phylogenetic tree). From these 26 studies we extracted 205 effect sizes (based on 1096 control and 1132 treatment subjects), expressed as Cohen’s *d,* calculated as:

$$ d=\frac{{\overline{x}}_1 - {\overline{x}}_2}{\mathrm{s}} $$

where ${\overline{x}}_1$ represents the mean value of the reproductive measure for the control group, ${\overline{x}}_2$ represents the mean for the treatment group and s represents the pooled standard deviation (for s calculation see Additional file 1: Dialog S1).

### Moderators

In meta-analyses, the use of moderators (e.g. the effect of sex) is often required to explain variation in the effect across studies (heterogeneity [30], see Additional file 1: Dialog S1). Therefore, we extracted and examined the effect of the following moderators: (1) model species or not, (2) sex, (3) degree of restriction, (4) cost of reproductive trait (see below) and (5) type of control feeding (*Ad libitum* or 100 % feeding). As a result of the wide variety of reproductive measures taken, we attempted to categorise reproductive traits based on how much of the total cost of reproduction they were likely to represent. Reproductive traits were classified as: low cost, moderate cost or high cost (i.e., on an ordinal scale, see Additional file 1: Table S1). This measure of cost was graded to take into account species and sex specific costs. For example, in male *D. melanogaster*, ejaculate production was classified as low cost, courtship for a single mating event as medium cost and lifetime courtship investment as high cost. Although subjective, we feel the use of three categories allowed reasonably accurate assignment of traits to a particular category and was necessary to assess how many studies allowed individuals to experience near total reproductive costs. Furthermore, when categorising the cost of trait, we took the study species into consideration, to account for differences in reproductive biology between different species and particularly differences between vertebrate and invertebrate reproductive biology. This also enables cross species comparison, despite the wide variety of reproductive traits being measured.

### Statistical analysis

Analysis was carried out in R [31] using the packages *metaphor* [32] and *MCMCglmm* [33] implementing multi-level meta-analysis (MM) and phylogenetic multi-level meta-analytic models (PMM) [34, 35] (see Additional file 1: Dialog S1 for details). We first ran models without moderators to examine overall patterns and to compare phylogenetic and non-phylogenetic models. We then added single moderators to the models to examine their effects in isolation. Finally, we constructed a full model including all moderators of interest. In the results section, we present mean standardized difference between control and restricted groups, standard errors and 95 % credible intervals (CIs). When comparing phylogenetic models to non-phylogenetic models we present the Akaike information criterion (AIC), which is a model selection index, with the better model having a smaller AIC. Publication bias was examined through visual assessment of the data and through Eggers regression.

## Results and discussion

### Does DR reduce reproduction universally?

DR on average resulted in a significant reduction in reproduction (mixed-effect meta-analysis, MM: β _[meta-analytic mean]_ = −0.841, 95 % Confidence Intervals (CI) = [−1.374 to −0.308]). This effect remained robust even when the phylogenetic non-independence of the samples was accounted for (phylogenetic mixed effect meta-analysis, PMM: β _[meta-analytic mean]_ = −0.841, CI = [−1.374, −0.308], Additional file 1: Table S2). However, there was no evidence of a strong phylogenetic signal (*I* ^*2*^_[*phylogeny*]_ < 0.001 %, Additional file 1: Table S3) in the effect of DR on reproduction, suggesting a consistent pattern across taxa. Although the model including phylogenetic signal was a better fit by AIC score (phylogenetic AIC = 577.33, non-phylogenetic = 579.86), the improvement was small and was not true for the model including all moderators (see below). To facilitate comparison we present models without phylogenetic signal included from here onwards; results are qualitatively the same for models including phylogenetic signal. Despite the small phylogenetic signal, we observed high heterogeneity amongst studies (*I* ^*2*^_[*total*]_ *=* 98.65 %, Additional file 1: Table S3), suggesting that the reduction in reproduction in response to DR was more apparent in certain studies. As stated above, such large heterogeneity (*sensu* [30]) calls for the use of moderators in our models to try to explain variation among studies.

### Is there an effect of restriction severity?

**Fig. 2:**
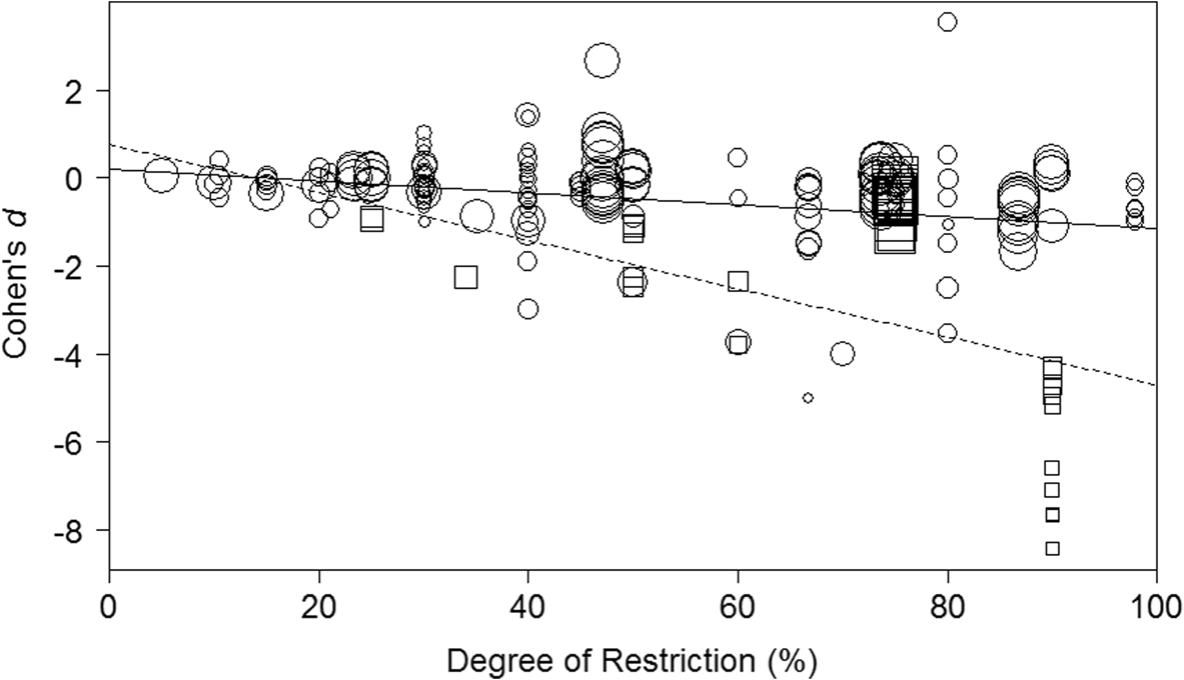
The effect of degree of restriction on effect size in model and non-model species. Effect sizes are Cohen’s *d*, the standardised mean difference in reproduction between the control and restricted groups (see Methods and Additional file 1: Dialog S1). Model species are represented by squares and the dashed line. Non-model species are represented by circles and solid line. Model species suffer a greater rate of decline in reproduction with increasing degree of restriction. Point sizes indicate the variance in the estimate of the effect size. Details of statistics are given in the main text

As discussed above, an obvious pattern to explore is how reproduction responds to variation in the degree of restriction applied. In general, increasingly severe restrictions appear to increase the lifespan extension achieved by DR, up to the point of malnutrition. However, a linear change in reproduction is not predicted by existing evolutionary theories of DR. We tested these predictions by fitting both a linear and quadratic effect of the degree of restriction. We found a linear negative effect of the degree of restriction (BMM: β _[Restriction]_ = -0.0158, CI = [−0.0219, −0.0096], Fig. 2, Additional file 1: Table S4), but no significant quadratic effect (MM: β^2^_[Restriction]_ = -0.884, CI = [−0.925, 2.694], Additional file 1: Table S4). This result is intriguing as it is counter to the predictions of both current evolutionary theories of DR [12, 29, 36]. One possible explanation for our inability to detect any non-linear pattern is a lack of data at particular restriction levels. Although many of the results analysed here were from studies with reasonably severe dietary restrictions (41 effect sizes, out of 205, with restriction levels greater than 75 % of *ad libitum*), there are very few data points with dietary restriction at *very* low or *very* high levels, particularly in model species (Fig. 2).

### Is there a model species effect?

**Fig. 3:**
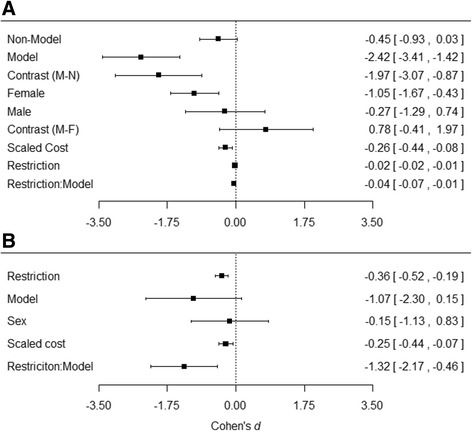
Forest plots showing effect sizes (Cohen’s *d*, standardised mean difference in reproduction between the control and restricted groups (see Methods and Additional file 1: Dialog S1)) of key moderators for the effect of dietary restriction (DR) on reproduction. Each point represents the Cohen’s *d* value with the 95 % credible intervals (CIs). Panel **a** represents the outputs from univariate models, with each moderator fitted individually. Each moderator subgroup (e.g. model or non-model species) is represented by a single point. Contrasts represent the difference between effect sizes of the subgroups (e.g. the difference between model (M) and non-model (N) species). Restriction:Model, represents the interaction between degree of restriction (%) and model or non-model species. Panel **b** shows the output from our full model accounting for all moderators, with each point representing the effect size for that moderator

A recent meta-analysis demonstrated that DR is nearly twice as effective at extending life in model compared to non-model species [1]. We therefore tested whether such a model species effect was also apparent for reproduction. To allow direct comparison, we defined model species as the same five species used in the meta-analysis on lifespan [1] (*i.e.*, *R. norvegicus*, *M. musculus*, *D. melanogaster*, *C. elegans*, *S.cerevisiae*). Our results show that model species suffer a statistically significant reduction in reproduction (MM: β _[model]_ = −2.42, CI = [-3.41, -1.43], Fig. 3a, Additional file 1: Table S5), whereas the reduction in non-model species was lower and marginally non-significant (MM: β _[non-model]_ = −0.445, CI = [−0.926, 0.033], Fig. 3a, Additional file 1: Table S5). Comparing these effects, DR had a significantly stronger effect on reproduction in model than non-model organisms (MM: β _[non-model/model difference]_ = -1.97, CI = [−3.07, −0.87], Fig. 3a, Additional file 1: Table S5).

In an attempt to disentangle this effect further, we included the interaction between model organism and degree of restriction. This analysis revealed a statistically significant interaction (MM: β _[restriction * model]_ = −0.0415, CI = [−0.0710, 0.0120], Figs. 2 and 3a, Additional file 1: Table S6); the rate of decline of reproduction with increasing DR was steeper in model than non-model species, suggesting that reproduction in model species is more responsive to resource availability than reproduction in non-model species. These results fit well with the findings of Nakagawa *et al.* [1] and with the disposable soma theory of the effect of DR on longevity, if this increased reduction in reproduction results in more resources being available for reallocation to somatic maintenance. However, the obvious question becomes why do model species have a greater reproductive response to increasing restriction than non-model species?

One possibility is that this is an unintentional effect of selection and subsequent adaptation to the laboratory environment [37]. For example, the laboratory environment is nutrient rich compared to the natural environment and selects for high fecundity but not longevity [38, 39]. Such an environment may inadvertently favour individuals that have greater plasticity in reproduction in response to nutrient availability. If such plasticity is maintained, either because it has no cost under laboratory conditions or because laboratory conditions vary enough to maintain plasticity, populations that have undergone generations of laboratory selection would be predicted to respond more plastically to food availability than populations that had not undergone such selection. On the other hand, natural environments may be predicted to be more variable than laboratory environments, particularly in food availability and this may be expected to select for increased plasticity in non-model species. Although a small number of studies compare the effectiveness of DR in extending lifespan in laboratory maintained populations versus wild or wild derived populations [37, 38, 40], results are inconsistent. It would therefore be interesting to increase the number of these studies and to use a range of food availabilities (rather than just two) to test whether laboratory populations are more plastic to food availability than wild derived populations. If so, inadvertent laboratory selection for high fecundity in a novel environment may have accounted for this plasticity.

Another possible explanation for the increased reproductive response to nutrient restriction in model species is that researchers can more effectively implement restriction in model species [1]. Model species have been studied in laboratory environments for many generations and thus diets are more likely to be optimised. In non-model species, where we know less about their nutritional requirements, “*ad libitum*” treatments may actually be fed to excess and foods are unlikely to be optimised. Thus when applying DR, the restricted group may be under much lower restriction levels than expected in non-model species. For example, a 75 % restriction may actually contain 90 % of the nutrients needed. Furthermore, the application of the geometric framework of nutrition to DR studies [41, 42], has provided a growing body of evidence that specific diet composition affect lifespan and reproduction and that this may be as, or even more, important than classical restriction (e.g. [2, 5, 27, 28]). Studies that use the same species may utilize diets with slightly different composition, which would undoubtedly affect results. It stands to reason, however, that model species which are frequently studied, will have better defined nutrient requirements and therefore that there may be less variation in diet composition and more consistent results. Obviously other explanations are possible, but our results and those of Nakagawa et al. [1] highlight the need for more research to investigate the cause of this model organism effect and how it may affect the generality of the conclusions drawn from investigations of DR.

### Is there sexual dimorphism?

We next addressed whether there are sex differences in the reproductive response to DR, similar to those observed in the longevity response [1]. Our analysis revealed that females suffer a significant reduction in reproduction under DR (MM: β _[female]_ = −1.05, CI = [−1.67, −0.43], Fig. 3a, Additional file 1: Table S7), but that this reduction is much smaller and statistically non-significant in males (MM: β _[male]_ = −0.274, CI = −1.291, 0.742, Fig. 3a, Additional file 1: Table S7). However, when comparing the magnitude of the effect between the sexes, we found no statistically significant difference between males and females (MM: β _[male/female difference]_ = 0.776, CI = [−0.414,1.967], Fig. 3a, Additional file 1: Table S7). The lack of statistical significance in comparison between the sexes is probably because of a lack of statistical power, with the sample size for males being particularly small, only 42 out of 205 effect sizes. These effect size estimates in males come from seven studies, covering five species, all of which were vertebrates (two bird species, one rodent, one primate and one fish species). The remaining studies were on females and there were no studies that allowed side-by-side comparisons of the effect of DR on males and females of the same species. Thus, studies that allow such direct comparison and generally more studies investigating DR in males would be desirable avenues of future research.

### Does the cost of the reproductive trait measured matter?

It seems intuitive that traits which are more costly or encompass a greater proportion of total reproductive investment, such as lifetime egg production, will suffer a greater reduction under DR than low cost traits, such as producing a single ejaculate. We therefore included the estimated costliness of the reproductive trait as a moderator. High and moderate cost reproductive traits were statistically significantly reduced under DR (MM L: β _[high]_ = −1.12, CI = [−1.71, −0.54]; β _[moderate]_ = −1.05, CI = [−1.62, −0.48], Additional file 1: Figure S2 and Table S8). In contrast, low cost traits suffered a much smaller and statistically non-significant reduction under DR (MM: β _[low]_ = −0.244, CI = [-0.861, 0.374], Additional file 1: Figure S2 and Table S8). This result is unsurprising, but has implications for future DR studies. If, as the disposable soma theory of DR suggests, the effect on longevity is due to a decrease in reproduction, future experiments must allow both control and restricted individuals to experience and express high cost reproductive traits. Otherwise, if individuals are only exposed to a small proportion of the costs of reproduction, the differences between control and restricted individuals are expected to be smaller and more difficult to detect. This may be one explanation for the current sex difference in the effect of DR if females are exposed to more of the costs of reproduction than males (see also below).

This point becomes particularly relevant when examining the current data set in detail. As mentioned above, our search criteria resulted in only 42 effect sizes for males versus 163 for females. Of these 42, only 1 was classed as a high cost reproductive trait (a measure combining all reproductive behaviour into a single score of sexual activity), 18 were moderate cost and the remaining 23 were low cost. The distribution for female traits was: 77 high cost, 69 moderate costs and 17 low cost traits. Given the difference in distribution of the cost categories between males and females (χ^2^_2df_ = 51.30, *p* < 0.001), it is unclear if the above sex differences in the reproductive response to DR are real or simply reflect difference in the costs of traits that have tended to be measured in males and females. To test this we fitted a final, ‘full’ model, to assess the effect of the inclusion of all moderators considered on the estimated effects.

### Putting it all together

When accounting for all of the individual moderators and the interaction between model species and the degree of restriction, the degree of restriction, the cost of the trait and the interaction were all statistically significant predictors of the reduction in reproduction under DR (MM: β _[Restriction]_ = −0.357, CI = [−0.520, −0.194]; β _[cost]_ = -0.252, CI = [−0.436, −0.067]; β _[restriction : model]_ = −1.32, CI = [−2.17, −0.47], Fig. 3b, Additional file 1: Table S9). This model had a conditional *R* ^2^ value of 78.8 % with random effects explaining 33.2 % and fixed effects explaining 45.6 % of the variation in effect size between studies [43]. When the interaction between model species and restriction was removed, restriction, model species and cost of trait remained as significant predictors (Additional file 1: Table S10).

As with the initial models, we also fitted models that accounted for the phylogenetic non-independence of species, with the non-phylogenetic model being the better fit (including interaction, phylogenetic AIC = 530.08, non-phylogenetic AIC = 528.08 (Additional file 1: Tables S9 and S11); excluding interaction, phylogenetic AIC = 539.22, non-phylogenetic AIC = 537.22 (Additional file 1: Tables S10 and S12)). This result suggests that the reduction in reproduction observed under DR is robust and phylogenetically conserved (*I* ^2^_*[phylogeny]*_ < 0.001 % Additional file 1: Table S13), but that the rate of reduction is greater in model species compared to non-model species. Furthermore, the reduction in reproduction was greater when examining more costly traits. Of particular interest when fitting the full model was the effect of including the cost of the trait on the sex difference in the effect of DR. When accounting for all other moderators, the difference between males and females was reduced (MM: β _[male/female difference]_ = −0.151, CI = [−1.132, 0.830] compared to MM: β _[male/female difference]_ = 0.776, CI = [−0.414, 1.967] in the model only containing sex, Fig. 3a and b). This result implies that the supposed sex differences in response to DR are being driven by experimental design, particularly the costs of reproduction experienced by the sexes.

Essential for all meta-analyses is the assessment of potential publication bias, as interpretation of results of meta-analyses assumes minimal publication bias in the literature [44]. Visual assessment of our data showed no obvious sign of publication bias (Additional file 1: Figure S3). Furthermore, statistical assessment revealed no significant publication bias in our data set once accounting for heterogeneity [35] (Eggers regression on the ‘meta-analytic’ residuals; β _[intercept]_ = 0.0780, S.E. = 0.0778, *p* = 0.317).

## Conclusions

Our results represent the first formal meta-analysis of the effect of DR on reproduction, an important issue given some studies suggesting the effect of DR on longevity can be achieved independently of reproduction [17]. Above, we present three main findings that suggest explanations for outstanding issues in this field and avenues for future research. First, DR does lead to a reduction in reproduction but, in line with longevity [1], this effect is stronger in model species. We discuss a number of possible explanations for this phenomenon. However, it is clear more studies are needed as any bias in patterns from model species as a result of laboratory adaptation have far reaching consequences for the role of DR studies in understanding and mitigating ageing and its application to humans [3]. Second, reproduction declines linearly with increasing DR, at odds with both current evolutionary theories of DR [12, 29, 38]. It is possible that our failure to detect a non-linear response of reproduction to DR was due to a lack of data at certain levels of restriction. More work across a broader range of restriction levels is needed to improve our power to detect non-linear effects and thus assess and compare alternative evolutionary hypotheses on DR effects [45, 46].

Finally, although our results support a sex difference in the response of reproduction to DR, they suggest this may be due to males and females being exposed to different levels of reproductive costs in the majority of experiments. An alternative explanation is that the longevity-reproduction trade-off can be uncoupled, with diets that maximize longevity not necessarily minimizing reproduction and that this effect can be sex specific [2, 28]. Definitive conclusions are difficult to draw because relatively few studies investigate the effect of DR on reproduction in males or allow direct comparison of males and females in the same study using a range of diets (but see [2, 28]). This is presumably because of the difficulty of designing meaningful measures of male reproductive investment that would encompass the majority of the costs. One potential solution is to measure many male reproductive traits and combine them into an overall score of reproductive investment [47]. Even if this is not possible, future DR studies must carefully consider the biology of the study organism and ensure both sexes are exposed to as close to the complete costs of reproduction as possible. For males this will usually include allowing costs such as those incurred while attracting females and direct competition with other males. By doing such experiments, we can start to assess whether sex differences in the response to DR, both in terms of reproduction and longevity, are a real and interesting sexual dimorphism, or an artefact of experimental design.

## Additional file

Additional file 1: Further information is provided in Additional file 1.doc, which contains more detailed methods, supplementary figures and supplementary tables. (DOCX 100 kb)

## References

[1] Nakagawa S, Lagisz M, Hector KL, Spencer HG (2012). Comparative and meta-analytic insights into life extension via dietary restriction. Aging Cell.

[2] Jensen K, McClure C, Priest NK, Hunt J (2015). Sex‐specific effects of protein and carbohydrate intake on reproduction but not lifespan in *Drosophila melanogaster*. Aging Cell.

[3] Selman C (2014). Dietary restriction and the pursuit of effective mimetics. Proc Nutr Soc.

[4] Jiang JC, Jaruga E, Repnevskaya MV, Jazwinski SM (2000). An intervention resembling caloric restriction prolongs life span and retards aging in yeast. FASEB J.

[5] Lakowski B, Hekimi S (1998). The genetics of caloric restriction in *Caenorhabditis elegans*. Proc Natl Acad Sci U S A.

[6] Lee KP, Simpson SJ, Clissold FJ, Brooks R, Ballard JW, Taylor PW, Soran N, Raubenheimer D (2008). Lifespan and reproduction in *Drosophila*: New insights from nutritional geometry. Proc Natl Acad Sci U S A.

[7] Simons MJP, Koch W, Verhulst S (2013). Dietary restriction of rodents decreases aging rate without affecting initial mortality rate – a meta-analysis. Aging Cell.

[8] Colman RJ, Beasley TM, Kemnitz JW, Johnson SC, Weindruch R, Anderson RM (2014). Caloric restriction reduces age-related and all-cause mortality in rhesus monkeys. Nat Commun.

[9] Austad SN (1989). Life extension by dietary restriction in the bowl and doily spider, *Frontinella pyramitela*. Exp Gerontol.

[10] Terzibasi E, Lefrançois C, Domenici P, Hartmann N, Graf M, Cellerino A (2009). Effects of dietary restriction on mortality and age‐related phenotypes in the short‐lived fish *Nothobranchius furzeri*. Aging Cell.

[11] Kirkwood TBL (1977). Evolution of ageing. Nature.

[12] Shanley DP, Kirkwood TBL (2000). Calorie restriction and aging: a life-history analysis. Evolution.

[13] Williams GC (1966). Natural selection, the costs of reproduction, and a refinement of Lack’s principle. Am Nat.

[14] Chippindale AK, Leroi AM, Kim SB, Rose MR (1993). Phenotypic plasticity and selection in *Drosophila* life-history evolution. I. nutrition and the cost of reproduction. J Evol Biol.

[15] Chapman T, Partridge L (1996). Female fitness in *Drosophila melanogaster*: an interaction between the effect of nutrition and of encounter rate with males. Proc R Soc B.

[16] Ball ZB, Barnes RH, Visscher MB (1947). The effects of dietary caloric restriction on maturity and senescence, with particular reference to fertility and longevity. Am J Physiol.

[17] Mair W, Sgro CM, Johnson AP, Chapman T, Partridge L (2004). Lifespan extension by dietary restriction in female *Drosophila melanogaster* is not caused by a reduction in vitellogenesis or ovarian activity. Exp Geront.

[18] Leroi MA (2001). Molecular signals versus *Loi de Balancement*. Trends Ecol Evol.

[19] Kaitala A (1987). Dynamic life-history strategy of the waterstrider *Gerris thoracicus* as an adaptation to food and habitat variation. Oikos.

[20] Boggs CL, Ross CL (1993). The effect of adult food limitation on life history traits in *Speyeria Mormonia* (Lepidoptera: Nymphalidae). Ecology.

[21] Inness CL, Metcalfe NB (2008). The impact of dietary restriction, intermittent feeding and compensatory growth on reproductive investment and lifespan in a short-lived fish. Proc R Soc B.

[22] Burger JMS, Promislow DEL. Sex-specific effects of interventions that extend fly life span. Sci Aging Knowl Environ. 2004:pe30. doi:10.1126/sageke.2004.28.pe30. 10.1126/sageke.2004.28.pe30 15254318

[23] Cooper TM, Mockett RJ, Sohal BH, Sohal RS, Orr WC (2004). Effect of caloric restriction on life span of the housefly, *Musca domestica*. FASEB J.

[24] Magwere T, Chapman T, Partridge L (2004). Sex differences in the effect of dietary restriction on life span and mortality rates in female and male *Drosophila Melanogaster*. J Gerontol A Biol Sci Med Sci.

[25] Bonduriansky R, Maklakov A, Zajitschek F, Brooks R (2008). Sexual selection, sexual conflict and the evolution of ageing and life span. Funct Ecol.

[26] Vinogradov AE (1998). Male reproductive strategy and decreased longevity. Acta Biotheor.

[27] Carey JR, Harshman LG, Liedo P, Muller HG, Wang JL, Zhang Z (2008). Longevity–fertility trade-offs in the tephritid fruit fly, *Anastrepha ludens*, across dietary-restriction gradients. Aging Cell.

[28] Maklakov AA, Simpson SJ, Zajitschek F, Hall MD, Dessmann J, Clissold F, Raubenheimer D, Bonduriansky R, Brooks RC (2008). Sex-specific fitness effects of nutrient intake on reproduction and lifespan. Curr Biol.

[29] Adler MI, Bonduriansky R (2014). Why do the well-fed appear to die young?. Bioessays.

[30] Higgins JP, Thompson SG, Deeks JJ, Altman DG (2003). Measuring inconsistency in meta-analyses. BMJ.

[31] R Core Team. R: A language and environment for statistical Computing. Vienna, Austria. 2016. https://R-project.org/.

[32] Viechtbauer W (2010). Conducting meta-analyses in R with the metafor package. J Stat Softw.

[33] Hadfield JD (2010). MCMC methods for multi-response generalized linear mixed models: the MCMCglmm R package. J Stat Softw.

[34] Hadfield J, Nakagawa S (2010). General quantitative genetic methods for comparative biology: phylogenies, taxonomies and multi‐trait models for continuous and categorical characters. J Evol Biol.

[35] Nakagawa S, Santos ES (2012). Methodological issues and advances in biological meta-analysis. Evol Ecol.

[36] Mitteldorf J (2001). Can experiments on caloric restriction be reconciled with the disposable soma theory for the evolution of senescence?. Evolution.

[37] Harper JM, Leathers CW, Austad SN (2006). Does caloric restriction extend life in wild mice. Aging Cell.

[38] Miller RA, Harper JM, Dysko RC, Durkee SJ, Austad SN (2002). Longer life spans and delayed maturation in wild-derived mice. Exp Biol Med.

[39] Austad SN, Kristan DM (2003). Are mice caloric restricted in nature. Aging Cell.

[40] Metaxakis A, Partridge L (2013). Dietary restriction extends lifespan in wild-derived populations of *Drosophila melanogaster*. PLoS One.

[41] Simpson SJ, Raubenheimer D (2007). Caoloric restriction and aging revisited: the need for a geometric analysis of the nutritional bases of aging. J Gerontol A Biol Sci Med Sci.

[42] Simpson SJ, Raubenheimer D (2009). Macronutrient balance and lifespan. Aging.

[43] Nakagawa S, Schielzeth H (2013). A general and simple method for obtaining R2 from generalized linear mixed‐effects models. Methods Ecol Evol.

[44] Egger M, Smith GD, Schneider M, Minder C (1997). Bias in meta-analysis detected by a simple, graphical test. BMJ.

[45] Tatar M (2011). The plate half-full: status of research on the mechanisms of dietary restriction in *Drosophila melanogaster*. Exp Gerontol.

[46] Flatt T (2014). Plasticity of lifespan: a reaction norm perspective. Proc Nutr Soc.

[47] Devigili A, Kelley JL, Pilastro A, Evans JP (2013). Expression of pre- and postcopulatory traits under different dietary conditions in guppies. Behav Ecol.

