# Supplementary material for: The effect of dietary restriction on reproduction: a meta-analytic perspective
Source: BMC Evol Biol. 2016 Oct 7;16:199. doi: 10.1186/s12862-016-0768-z (PMC5054627; doi:10.1186/s12862-016-0768-z)
Supplement: Additional file 1: — Further information is provided in Additional file 1.doc, which contains more detailed methods, supplementary figures and supplementary tables. (DOCX 100 kb) [file 12862_2016_768_MOESM1_ESM.docx]

**Dialog S1**

**Collecting studies on dietary restriction (DR) and reproduction.**

The data for the meta-analysis were collected through a search of ISI Web of Science and Scopus during December 2013 by J. P. Moatt using the search string ‘diet*/calor* + restriction + reproduction/ fertility/fecundity’. Backward and forward searching was carried out to identify additional papers that were missed in the main database search, as well the authors’ own literature collections on the subject were considered. Authors of interest were contacted in attempt to obtain unpublished data for inclusion in the analysis. However, no unpublished data matching the selection criteria were found. Grey literature and non-English language papers were also considered during selection. Of the 1,679 unique papers the search returned, papers were selected which had applied DR and reported some measure of reproduction, for treated (DR) and control females or males (usually presented as a means and standard errors). Papers were included if they met the following criteria:

1. Papers must be original empirical data using real animals, not reviews or computer simulations.
2. Animals must not be mutant or transgenic.
3. Degree of dietary restriction must be explicitly stated.
4. Intermittent feeding is allowed, as long as fasting period does not exceed the equivalent of every other day feeding. Feeding days must not allow compensatory gorging.
5. Information on the control groups intake must be given, and be either *ad libitum* or 100%.
6. Restriction must have been initiated prior to copulation and must remain constant throughout the course of the experiment.
7. There were no other confounding cofactors, such as resveratrol or pathogen treatment.

Additionally, we excluded studies where only measures of reproductive hormone levels were reported or information necessary for calculating effect sizes was missing (e.g. sample sizes, variances). Screening was carried out by J. P. Moatt between January and June 2014. Although the screening was carried out alone, discussion over the inclusion of a number of papers took place between C. A. Walling and J. P. Moatt.

**Extracting effect size**

In the majority of papers, reproductive data was presented in the main text as mean and standard error as well as sample sizes. In studies where this was not the case, authors were contacted in an attempt to obtain the relevant data. Effects sizes were then calculated using an effect size calculator [1]. Effect sizes are the standardised mean difference (SMD) Cohen’s *d*, a measure of the difference in reproduction between the control and restricted groups, standardised by the pooled standard deviation estimates from the two groups.

$$d=\frac{\overline{x}_{1}- \overline{x}_{2}}{s}$$

X_1_ = mean for control group

X_2_ = mean for treatment group

s = pooled standard deviation. Calculated as below:

$$s=\sqrt{\frac{\left( n_{1}-1 \right)s_{1}^{2}+(n_{2}-1)s_{2}^{2}}{n_{1}+ n_{2}-2}}$$

n_1_ = sample size of control group

n_2_ = sample size of treatment group

s_1_ = standard deviation of control group

s_2_ = standard deviation of treatment group

**Extracting Moderators (DR associated variables)**

Methods sections from each paper were examined and any relevant moderators were extracted and recorded as follows:

- Model Species: 1 = yes, 0 = no, model species counted as the same five model species as in Nakagawa *et. al.* [2]: yeast (*Sacchromyces cerevisiae*), nematode (*Caenorhabditis elegans*), fruit fly (*Drosophila melanodaster*), mouse (*Mus musculus*) and rat (*Rattus norvegicus*).
- Strain name/type: unique strain names for a particular species (note that unique names are given for WT or the same strain names for different species).
- Sex: sex of the group *d* was extracted for (M = male, F = female).
- Food schedule: feeding regime used (D = daily, W = Weekly).
- Type of restriction being used: CNM = Calorie and nutrient manipulation, these were papers that included a number of diets of varied composition. However, these studies were only included if each diet was provided at multiple restriction levels, including a control level; FC = food concentration, where lower concentrations of the same food medium were used in treatment relative to control group; FS = feeding schedule, where restriction was implemented through a feeding schedule, as less frequent feeding than in the control group, e.g. every other day feeding vs. every day feeding; FW = food weight, where the same food was given in smaller quantities in treatment relative to control group.
- Feeding regime of control: 0 = 100% feeding, where individuals were given a set quantity and this was counted as fully fed; 1 = *ad libitum* where unrestricted access to food was allowed.
- Units of control and treatment group nutrition levels (when given): e.g., J/day/individual.
- Calories in control diet (when information provided): caloric density of the food.
- Costliness of the reproductive trait: A categorical measure that describes the degree to which the reproductive trait measured reflects the total cost of reproduction in the species used: 1 = low cost – trait represents a relatively small fraction of the total cost of reproduction in that species, 2 = moderate cost, trait represents a moderate fraction of the total cost of reproduction in that species, 3 = high cost, trait represents the majority of the cost of reproduction in that species. This measure accounted for differences between species and sexes within species. For example, in *D. melanogaster*, ejaculate production is classed as low cost, courtship for a single mating event represents a medium cost and lifetime courtship investment is high cost, as courtship is thought to be one of the most costly aspects of reproduction for male *D. melanogaster* [3]. For females, daily egg production represents a medium cost, whereas lifetime egg production is high cost, see Table S1.
- Reproductive measure examined: e.g., lifetime egg production, number of sperm.
- Units of the reproductive trait measured (where necessary): e.g., mass of eggs produced in g.
- The value of the reproductive trait being measured for the control group.
- Standard deviation of the mean for control group.
- Number of control individuals.
- Caloric value of restricted diet (when given).
- Restriction level, represented as a percentage decrease from control group: e.g. 40% restriction means treatment group give 60% of control diet.
- The value of the reproductive trait being measured for the restricted group.
- Standard deviation of the mean for restricted group.
- Number of restricted individuals.

Any other information considered relevant or important was noted. For complete records see Data S1 and for the detailed description of all the columns in the data table see Dialog S2.

**Constructing phylogenetic tree**

A topological (without branch lengths) phylogenetic tree was constructed for the subset of species included in this study using the Interactive Tree of Life (http://itol.embl.de/index.shtml). Polytomies among insect orders were resolved using information obtained from Trautwein *et al.* [4].

**General meta-analytic techniques**

For the main analyses we used mixed effects meta-analysis (MM) or phylogenetic mixed effects meta-analysis (PMM) implemented in the *metaphor* package [5], version 1.9-3, and *MCMCglmm* package [6] for R (R core team (2014)). As model results we present mean standardized difference between control and restricted groups, standard errors, and 95% credible intervals (CIs). When comparing phylogenetic models to non-phylogenetic models we present the Akaike information criterion AIC, which is a model selection index, with the better model having the smaller AIC. The *R* scripts for all analyses are available as supplementary materials with this article.

**Main meta-analytic models (Model 1 and 2)**

Models 1 and 2 (Table S2) were simple models only fitting the effect size as a response variable, with the intercept as the fixed factor and the following random factors; study ID, animal (species ID), group ID (identifies cases where multiple types of reproduction traits were reported for the same groups of individuals) and effect size ID. These were to account for the main sources of non-independence between our measures. Model 1 only differed from Model 2 in that it accounted for phylogenetic variance.

**Heterogeneity**

A meta-analysis will inevitably bring together studies that differ in design and set up, particularly in reference to treatments, exposures and outcomes explored, this is referred to as heterogeneity [7]. We must account for heterogeneity to explain the differences observed between the studies included in a meta-analysis. Here, we used an extended version of *I^2^* [7] as our heterogeneity statistic, which is described in Nakagawa and Santos [8]. This multi-level model extension of *I^2^* enables us to obtain heterogeneity due to each level or random factor.

**Meta-analytic models with moderators (Models 3-11)**

Our main question was to see whether investment in reproduction was decreased under DR. However, we also explored variables we thought may be important predictors of variation in the effect of DR on reproduction, known as moderators. We added each moderator separately to the main meta-analytical model (Model 2) to assess the effect of individual moderators (Models 3-7). These moderators included: (a) whether the control group was fed a specific pre-defined amount or concentration of food (100%) or were allowed *ad libitum* access to food (only included in full models 8 - 11), (b) whether the species was one of the five model species or not (Table S4, Model 3), (c) which sex was being studied (Table S5,Model 4), (d) the linear and quadratic effect of degree of restriction (Table S6, Model 5), (e) the relative cost of the reproductive trait being studied (low, moderate and high, Table S1 for trait classification, Table S7 for model output, Model 6). We also fitted the interaction between model/non-model species and degree of restriction (Table S8, Model 7). We then created a number of full models where all moderators were fitted at the same time (Tables S9-S13,Models 8 - 11). Models 8 and 9 included all moderators and the interaction between model/non-model species and degree of restriction. Models 10 and 11 included all moderators but excluded the interaction between model/non-model species and degree of restriction. Models 9 and 11 are models which account for the phylogenetic variance.

**Publication Bias**

Publication bias is the favouring of statistically significant results during publication, regardless of the underlying effect size. We used two typical ways of assessing publication bias: (1) visual inspection via a funnel plot and (2) Eggers regression, which assess bias through a regression method [9]. However, these methods assume that effect sizes are independent of each other. We therefore used meta-analytic residuals (sampling error and residuals) from our full model for Egger regression to fulfil this assumption. [8].


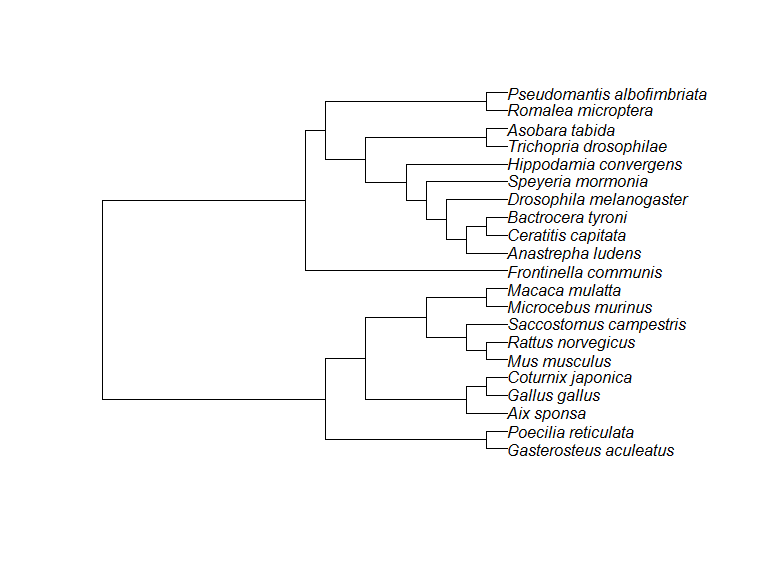
**Supplementary figures**

**Figure S1.** Phylogenetic tree of the 21 species used in the meta-analysis.


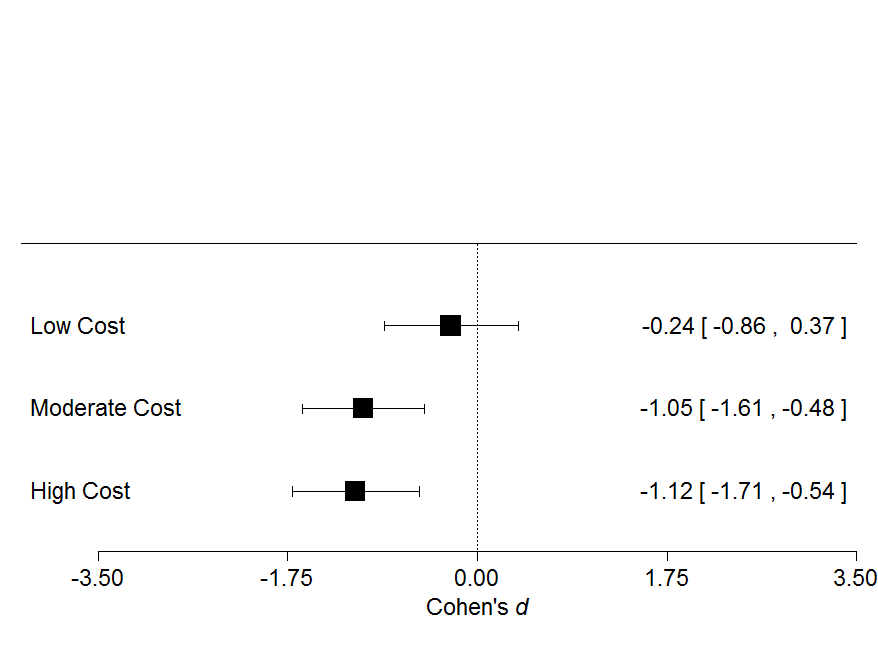


**Figure S2.** Forest plot showing effect sizes (Cohen’s *d*) for the effect of dietary restriction (DR) on reproduction, for different levels of cost of reproductive trait included as a moderator. Each point represents the Cohen’s *d* value for that moderator with the 95% credible intervals (CIs). High and moderate cost traits undergo a significant reduction under DR, however low cost traits do not.


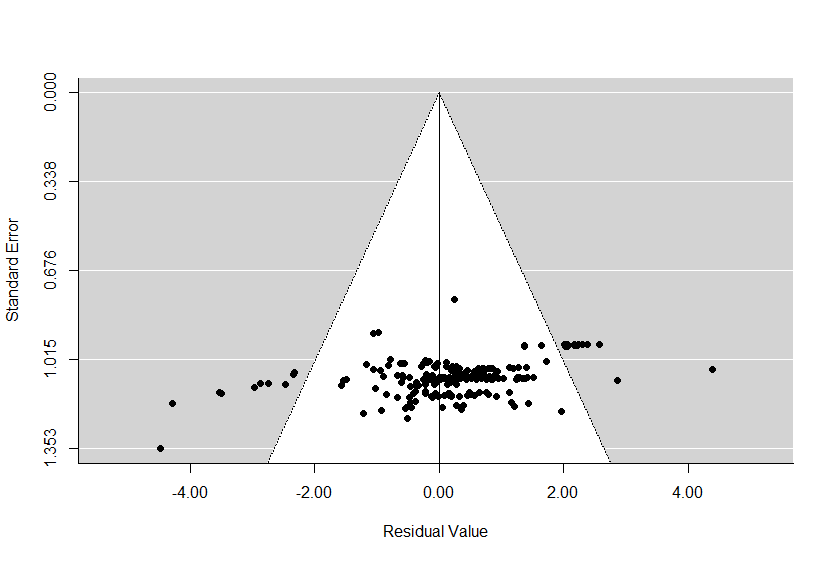


**Figure S3.** Funnel plot to allow visualisation of potential publication bias in our data set. The X axis represents the residual values from the non-phylogenetic mixed effects model containing all moderators and the interaction of restriction and model species, the Y axis represents the standard error. Publication bias indicated if data points clustered towards zero residual values as standard error decreases. Visual inspection suggests this is not the case.

**Supplementary Tables**

**Table S1** List of reproductive traits and the cost category they were assigned.

| **Low Cost** (n=40) | **Medium Cost** (n=87) | **High Cost** (n=78) |
| --- | --- | --- |
| Number of eggs fertilised (measured when only males under DR) | Testes weight, lifetime investment in sperm production | Number of females pregnant at least once in lifetime, lifetime investment in reproduction |
| Proportion of fertile eggs that hatch (measured when only males under DR) | Daily fecundity, high cost but not lifetime investment | Total fecundity, lifetime investment in egg production. |
| Pair formation when both sexes under DR, measured as proportion of birds that successfully pair | Size of 1^st^ egg clutch, similar to above, high cost but not lifetime investment. | Reproductive effort, lifetime measure |
| All sperm / ejaculate composition, e.g. sperm length, ejaculate volume, proportion of live sperm etc | Date of 1^st^ egg production, age of sexual maturity | Lifetime clutch production |
| Time per clutch, time to lay eggs | Gestation length, assuming more significant cost to female than litter growth/weight | Number of females reproducing during breeding season. |
| Mating-oviposition interval, not measuring number of eggs produced or matured in this time | Male courtship of females, known to be costly but only one reproductive behaviour measured | Sexual activity, measuring full range of male precopulatory behaviour |
| Foetal growth (g per day) | Egg load, females were unmated, killed and dissected.  Eggs counted midway through life |  |
| Litter body mass at birth | Reproductive success for single breeding season, not lifetime reproductive success |  |
| Egg mass, investment in single egg | Litter size, combination of egg number and provisioning of foetus |  |
|  | Number of clutches/eggs for part of life, not lifetime investment in eggs |  |
|  | Reproductive period (days), measure of single reproductive season |  |
|  | Oviposition days for single breeding season |  |
|  | Reproductive success, single breeding season |  |

**Table S2** Comparing phylogenetic mixed effect model (PMM, Model 1) and non-phylogenetic mixed effect model (MM, Model 2) estimates of the effect of DR on reproduction. AIC taken from ML models.

|  | Effect size | SE | Lower CI | Upper CI | AIC |
| --- | --- | --- | --- | --- | --- |
| PMM | -0.841 | 0.272 | -1.374 | -0.308 | 577.33 |
| MM | -0.841 | 0.272 | -1.374 | -0.308 | 579.86 |

**Table S3** Table of heterogeneity statistics (*I*^2^ values) for Models 1 and 2.

|  | Model 1 | Model 2 |
| --- | --- | --- |
| Total Heterogeneity | 98.65 | 98.65 |
| Variance due to Phylogeny | 0.0000667 | NA |
| Variance due to Study | 74.83 | 74.83 |
| Variance due to Group | 3.91 | 3.91 |
| Residuals against sampling error | 19.91 | 19.91 |

**Table S4** Estimated effect sizes from the non-phylogenetic mixed effect model with the linear and quadratic effect of restriction as moderators (Model 5)

|  | Effect size | SE | Lower CI | Upper CI |
| --- | --- | --- | --- | --- |
| Restriction | -0.016 | 0.003 | -0.022 | -0.010 |
| Restriction^2^ | 0.884 | 0.923 | -0.925 | 2.694 |

**Table S5** Estimated effect sizes from the non-phylogenetic mixed effect model with model/non-model species fitted as a moderator (Model 3).

|  | Effect size | SE | Lower CI | Upper CI |
| --- | --- | --- | --- | --- |
| Model | -2.416 | 0.506 | -3.406 | -1.425 |
| Non-model | -0.447 | 0.245 | -0.926 | 0.033 |
| Contrast | -1.969 | 0.562 | -3.070 | -0.868 |

**Table S6** Estimated effect sizes from the non-phylogenetic mixed effect model with the interaction between model species and restriction fitted as moderators (Model 7)

|  | Effect size | SE | Lower CI | Upper CI |
| --- | --- | --- | --- | --- |
| Restriction | -0.013 | 0.003 | -0.020 | -0.007 |
| Model | 0.769 | 1.035 | -1.261 | 2.798 |
| Restricition:Model | -0.042 | 0.015 | -0.071 | -0.012 |

**Table S7** Estimated effect sizes from the non-phylogenetic mixed effect model with sex as a moderator (Model 4)

|  | Effect size | SE | Lower CI | Upper CI |
| --- | --- | --- | --- | --- |
| Female | -1.051 | 0.316 | -1.671 | -0.431 |
| Male | -0.274 | 0.519 | -1.291 | 0.742 |
| Contrast | 0.776 | 0.608 | -0.414 | 1.967 |

**Table S7** Estimated effect sizes from the non-phylogenetic mixed effect model with cost of trait fitted as a moderator (Model 6)

|  | Effect size | SE | Lower CI | Upper CI |
| --- | --- | --- | --- | --- |
| Low Cost | -0.244 | 0.315 | -0.861 | 0.374 |
| Moderate Cost | -1.050 | 0.288 | -1.615 | -0.484 |
| High Cost | -1.124 | 0.298 | -1.708 | -0.539 |

**Table S9** Estimated effect sizes from the non-phylogenetic mixed effect model with all moderators fitted, including the interaction between restriction and model species (Model 8). AIC taken from ML models.

|  | Effect size | SE | Lower CI | Upper CI |
| --- | --- | --- | --- | --- |
| Year | 0.034 | 0.018 | -0.001 | 0.067 |
| *Ad Lib* feeding | -0.173 | 0.434 | -1.024 | 0.678 |
| Restriction | -0.357 | 0.083 | -0.520 | -0.194 |
| Model species | -1.074 | 0.625 | -2.298 | 0.150 |
| Male | -0.151 | 0.501 | -1.132 | 0.830 |
| Scaled cost | -0.252 | 0.094 | -0.436 | -0.067 |
| Restricition:Model | -1.317 | 0.435 | -2.169 | -0.465 |

AIC = 528.08

**Table S10** Estimated effect sizes from the non-phylogenetic mixed effect model with all moderators fitted, omitting the interaction between restriction and model species (Model 10). AIC taken from ML models.

|  | Effect size | SE | Lower CI | Upper CI |
| --- | --- | --- | --- | --- |
| Year | 0.014 | 0.019 | -0.024 | 0.051 |
| *Ad Lib* feeding | 0.295 | 0.470 | -0.627 | 1.217 |
| Restriction | -0.390 | 0.084 | -0.554 | -0.226 |
| Model species | -1.634 | 0.685 | -2.977 | -0.291 |
| Male | -0.148 | 0.569 | -1.264 | -0.069 |
| Scaled cost | -0.257 | 0.096 | -0.446 | -0.054 |

AIC = 537.22

**Table S11** Estimated effect sizes from the phylogenetic mixed effect model with all moderators fitted, including the interaction between restriction and model species (Model 9). AIC taken from ML models.

|  | Effect size | SE | Lower CI | Upper CI |
| --- | --- | --- | --- | --- |
| Year | 0.034 | 0.018 | -0.001 | 0.070 |
| *Ad Lib* feeding | -0.173 | 0.434 | -1.024 | 0.679 |
| Restriction | -0.357 | 0.083 | -0.520 | -0.194 |
| Model species | -1.074 | 0.625 | -2.298 | 0.150 |
| Male | -0.151 | 0.501 | -1.133 | 0.830 |
| Scaled cost | -0.252 | 0.094 | -0.436 | -0.067 |
| Restricition:Model | -1.317 | 0.435 | -2.169 | -0.465 |

AIC = 530.08

**Table S12** Estimated effect sizes from the phylogenetic mixed effect model with all moderators fitted, omitting the interaction between restriction and model species (Model 11). AIC taken from ML models.

|  | Effect size | SE | Lower CI | Upper CI |
| --- | --- | --- | --- | --- |
| Year | 0.014 | 0.019 | -0.024 | 0.051 |
| *Ad Lib* feeding | 0.295 | 0.470 | -0.627 | 1.217 |
| Restriction | -0.390 | 0.084 | -0.554 | -0.226 |
| Model species | -1.634 | 0.685 | -2.977 | -0.291 |
| Male | -0.148 | 0.569 | -1.264 | 0.968 |
| Scaled cost | -0.257 | 0.096 | -0.446 | -0.069 |

AIC = 539.22

**Table S13** Table of heterogeneity statistics (*I*^2^ values) for Models 8 and 9.

|  | Model 8 | Model 9 |
| --- | --- | --- |
| Total Heterogeneity | 97.54 | 97.58 |
| Variance due to Phylogeny | NA | 0.00002 |
| Variance due to Study | 59.54 | 59.54 |
| Variance due to Group | 0.00006 | 0.00 |
| Residuals against sampling error | 38.04 | 38.03 |

**References.**

[1] Lipsey, M.W. & Wilson, D.B. 2001 *Practical meta-analysis*, Sage publications Thousand Oaks, CA.

[2] Nakagawa, S., Lagisz, M., Hector, K.L. & Spencer, H.G. 2012 Comparative and meta-analytic insights into life extension via dietary restriction. *Aging Cell* **11**, 401-409.

[3] Cordts, R. & Partridge, L. 1996 Courtship reduces longevity of male *Drosophila melanogaster*. *Anim. Behav.* **52**, 269-278.

[4] Trautwein, M.D., Wiegmann, B.M., Beutel, R., Kjer, K.M. & Yeates, D.K. 2012 Advances in insect phylogeny at the dawn of the postgenomic era. *Annu. Rev. Entomol*. **57**, 449-468.

[5] Viechtbauer, W. 2010 Conducting meta-analyses in R with the metafor package. *J. Stat. Softw*. **36**, 1-48.

[6] Hadfield, J.D. 2010 MCMC methods for multi-response generalized linear mixed models: the MCMCglmm R package. *J. Stat. Softw*. **33**, 1-22.

[7] Higgins, J.P. & Thompson, S.G. 2002 Quantifying heterogeneity in a meta-analysis. *Stat. Med*. **21**, 1539-1558.

[8] Nakagawa, S. & Santos, E.S. 2012 Methodological issues and advances in biological meta-analysis. *Evol Ecol* **26**, 1253-1274.

[9] Egger, M., Smith, G.D., Schneider, M. & Minder, C. 1997 Bias in meta-analysis detected by a simple, graphical test. *BMJ*. **315**, 629-634.
